# Salinity Negatively Impacts Protistan and Fungal Community Stability and Shapes Assembly Processes in Freshwater Ecosystems

**DOI:** 10.1111/1758-2229.70209

**Published:** 2025-10-05

**Authors:** Ivana Stanić, Andrea Čačković, Sandi Orlić

**Affiliations:** ^1^ Division of Materials Chemistry Ruđer Bošković Institute Zagreb Croatia; ^2^ Center of Excellence for Science and Technology‐Integration of Mediterranean Region (STIM) Zagreb Croatia; ^3^ University of Montenegro Podgorica Montenegro

**Keywords:** 18S rRNA gene sequencing, biogeography, ITS gene sequencing, neutral community model, salinity gradient, stohastic processes

## Abstract

In freshwater lakes, protistan and fungal communities play crucial roles in the microbial loop as bacterivorous consumers, facilitating nutrient cycling and maintaining microbial balance by controlling bacterial populations. However, understanding of their functional roles and community assembly across varying environmental gradients in different lake ecosystems remains limited. In this study, we used 18S rRNA gene amplicon sequencing and multivariate statistical analyses to investigate the spatiotemporal and biogeographical patterns of protistan and fungal communities in the water column of two different lake systems in Croatia. Our results revealed that these complex communities were dominated by Chlorophyta, Ciliophora and Cryptophyta as well as Ascomycota, Basidiomycota and Chytridiomycota. Null model analysis showed that stochastic processes dominated most of the prokaryotic and fungal communities across sampled lakes and fractions, with seasonally salty Lake Crniševo having more prominent variable selection due to the presence of a salinity gradient. Also, it was discovered that salinity had a negative influence on the stability of both protistan and fungal communities in Lake Crniševo, acting as a major selective pressure. These results provide valuable insights into the community stability and assembly mechanisms of protistan and fungal communities in lake ecosystems and their responses to environmental changes.

## Graphical abstract

We investigated the spatiotemporal and biogeographical patterns of protistan and fungal communities in the water column of two different lake systems in Croatia through almost 2 years. Salinity has a strong influence on both communities.

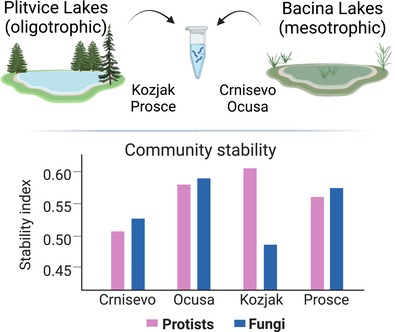

## 1 Introduction

Lake ecosystems host diverse protistan and fungal communities that play important roles within aquatic microbial food webs, acting as primary producers, organic matter decomposers and bacterivorous consumers (Gad et al. 2020; Xiao et al. 2024). They also contribute to nutrient recycling in freshwater ecosystems (Grossart et al. 2019). The structure and function of these communities are shaped by a combination of deterministic and stochastic processes that drive community assembly, such as environmental filtering, biotic interactions and dispersal limitation (Lindström and Langenheder 2012; Stegen et al. 2012). Understanding the assembly mechanisms and stability of these communities is crucial, as they are sensitive to environmental change, including nutrient load, temperature fluctuations and hydrological variations. Environmental filtering and competitive interactions can shape protistan community composition, where certain taxa may dominate under specific environmental conditions, leading to predictable patterns of assembly. Conversely, stochastic processes, such as random dispersal events or drift, introduce variability that can increase resilience and functional redundancy, promoting community stability in the face of disturbances (Biggs et al. 2020; Renes et al. 2020).

Research has shown that community stability in freshwater systems often depends on the balance between these assembly mechanisms, where a high degree of functional redundancy—common in fungal communities—may buffer the system against species loss (Shade et al. 2012). Additionally, the association of organisms with particulate matter (particle‐associated, PA) versus free‐living (FL) lifestyles can influence community dynamics, as particle association often provides protection from grazing and enhances local resource availability, potentially leading to greater stability in PA communities (Simon et al. 2002; Grossart 2010). By elucidating these assembly mechanisms, researchers can better understand the resilience of freshwater protistan and fungal communities, particularly in the context of increasing anthropogenic pressures.

The two studied lake ecosystems differ significantly in climate, trophic status, mixing patterns and surrounding vegetation. The Plitvice Lakes are located in a temperate continental climate with warm summers (Cfb climate classification), where the mean temperature of the coldest month is below 0°C, the warmest month is below 22°C and precipitation is evenly distributed year‐round (Šegota and Filipčić 2003). These lakes are oligotrophic and dimictic, with low nutrient and dissolved organic matter (DOM) content (Kajan et al. 2023) and high levels of dissolved oxygen (Miliša and Ivković 2023). Conversely, the Baćina Lakes lie within a hot‐summer Mediterranean climate (Csa), characterised by a mean temperature above 0°C in the coldest month, above 22°C in the warmest month and a summer dry season with less than 40 mm of precipitation in the driest month (Šegota and Filipčić 2003; Miko and Ilijanić 2015). These lakes are mesotrophic and monomictic, exhibiting summer stratification and winter mixing (Miko and Ilijanić 2015; Hanžek et al. 2021). The Baćina Lakes system is a cryptodepression, allowing seawater inflow due to rising sea levels and the semipermeable limestone bedrock that links the aquifer to the Adriatic Sea (Miko and Ilijanić 2015). The porous limestone and lower precipitation during the summer reduce groundwater levels, leading to seawater intrusion deeper into the aquifer (Alfarrah and Walraevens 2018; Srzić et al. 2020). This salinisation predominantly affects Lake Crniševo, where seawater enters through a bottom sinkhole and the Mindel underwater salty spring (Miko and Ilijanić 2015). Although connected to Lake Crniševo by a narrow channel, Lake Oćuša has notably lower salinity, suggesting limited water exchange between the two lakes (Bonacci and Roje‐Bonacci 2020).

In this study, we aim to identify the spatiotemporal patterns and assembly mechanisms of protistan and fungal communities in freshwater lakes by comparing particle‐associated and free‐living lifestyles. Using ITS and 18S rRNA gene amplicon sequencing combined with multivariate and null model analyses, we want to examine the relative contributions of deterministic and stochastic processes shaping these communities across contrasting environmental gradients in two Croatian lake systems. This approach will deepen understanding of microbial community stability and resilience in freshwater ecosystems under environmental change.

## 2 Materials and Methods

### 2.1 Study Area and Sampling

**FIGURE 1:**
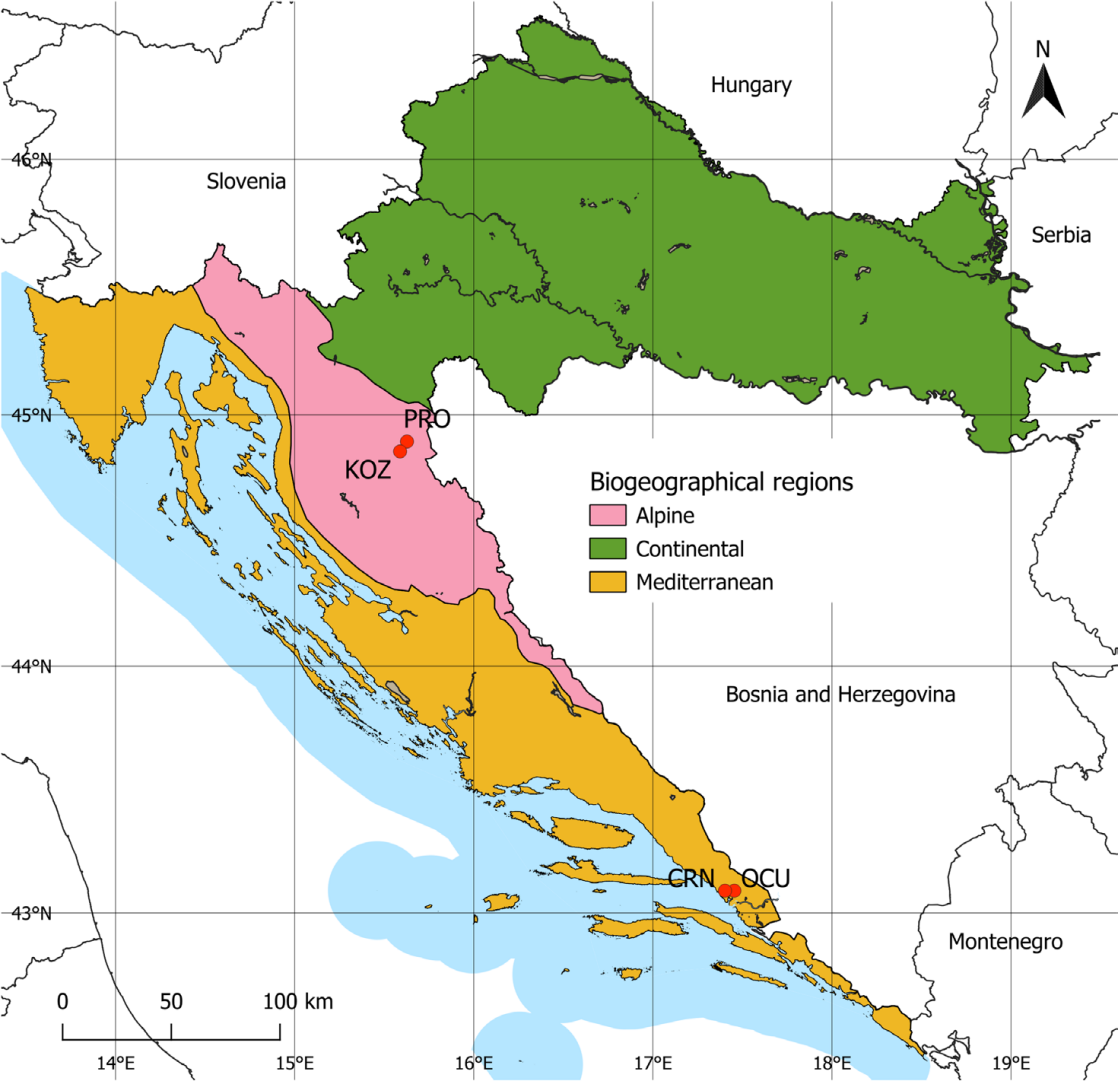
Study area with locations of the sampled lakes in the Alpine and Mediterranean biogeographical region. CRN, Crniševo Lake; KOZ, Kozjak Lake; OCU, Oćuša Lake; PRO, Prošće Lake.

Seasonal dynamics of protistan and fungal communities were examined in four lakes across two freshwater ecosystems in Croatia: the Plitvice Lakes and Baćina Lakes (Figure 1). Both systems consist of multiple interconnected lakes, with sampling concentrated on the largest ones—Lake Kozjak and Lake Prošće in the Plitvice Lakes system and Lake Crniševo and Lake Oćuša in the Baćina Lakes system. Water samples were collected monthly from four lakes, from May 2021 to February 2023 for Baćina Lakes and from June 2021 to September 2022 for Plitvice Lakes. Sampling depths were determined by the presence of a thermocline or oxycline, with a minimum of three depths samples when a thermocline was present (above, within and below the thermocline) and at least two depths when the water column was mixed (upper and bottom layers). In situ measurements of dissolved oxygen (DO), temperature, pH, fluorescent dissolved organic matter (fDOM), turbidity, conductivity and salinity were taken using an EXO3 Multiparameter Sonde (YSI, Yellow Springs, OH, USA). At each depth, 2‐l water samples were collected using a UWITEC water sampler (GmbH, Mondsee, Austria), transferred into sterile Nalgene PC bottles and immediately filtered through polycarbonate filters (Whatman Nuclepore Track‐Etch membrane, 47 mm diameter) with a 3 μm pore size for particle‐attached (PA) and a 0.2 μm pore size for free‐living (FL) microbial communities using a peristaltic pump (Masterflex, Cole‐Parmer, Vernon Hills, IL, USA). Filters were stored at −20°C until DNA extraction. The filtrate was further used for ion chromatography (Ca^2+^, Mg^2+^, K^+^, Na^+^, NH_3_^+^, SO_4_^2−^, Cl^−^, NO_3_^−^, PO_4_^3−^) with a Dionex ICS‐6000 DC ion chromatograph (Thermo Fisher Scientific, Waltham, MA, USA) and for dissolved organic carbon (DOC) analysis with a QBD1200 analyzer (Hach Company, Loveland, CO, USA).

### 2.2 Amplicon Sequencing and Processing

Total genomic DNA was extracted with the Dneasy PowerWater kit (Qiagen, Hilden, Germany) following the manufacturer's protocol. The V4 region of the 18S rRNA gene was amplified using the primer pair TAReuk454FWD1/TAReukREV3, following the protocol of Stoeck et al. (2010). The ITS2 region of the fungal rRNA gene was amplified with the primer pair ITS3‐Mix1‐Mix2 (TCCTCCGCTTAyTgATAtGc), a modified ITS3 Mix2 forward primer ITS3‐mkmix2 (CAWCGATGAAGAACGCAG) (Tedersoo et al. 2015) and the reverse primer ITS4, which is an equimolar mix of cwmix1 (TCCTCCGCTTAyTgATAtGc) and cwmix2 (TCCTCCGCTTAtTrATAtGc) (Wurzbacher et al. 2017). A unique dual‐barcoding two‐step PCR approach (UDB‐H12) was employed as outlined by Pjevac et al. (2021). Amplicons were sequenced in paired‐end mode (2 × 300 bp) on a MiSeq platform (Illumina, San Diego, CA, USA) at the Joint Microbiome Facility of the Medical University of Vienna and the University of Vienna. Details on sequence trimming and quality filtering settings can be found in Pjevac et al. (2021). Sequence data were processed in R (v.4.2.2, R Core Team 2024) using DADA2 according to the workflow by Callahan et al. (2016) in a pooled mode across all amplicon libraries per sequencing run. Taxonomic assignment was achieved by aligning 18S V4 ASV sequences against the PR2 reference database (v.4.12.0) (Guillou et al. 2013) and ITS2 ASV sequences against the UNITE reference database (v. 04.02.2020) (Nilsson et al. 2019). Functional groups for protistan ASVs were assigned based on taxonomy, categorising them as autotrophs, mixotrophs, parasites, osmotrophs and phagotrophs, according to Adl et al. (2019) and supporting literature (Reczuga et al. 2020; Suter et al. 2022). All statistical analyses and visualisations were performed using functions from the phyloseq (McMurdie and Holmes 2014), vegan (Oksanen et al. 2018), mgcv (Wood 2011), plspm (Sanchez et al. 2009) and ggplot2 (Wickham 2016) packages. Correlation analyses were evaluated at a significance threshold of *α* = 0.1 and relationships with *p* < 0.1 were considered statistically significant.

### 2.3 Neutral Model and Null Model Analyses

The neutral community model (NCM) (Sloan et al. 2006) was applied to evaluate the effects of stochastic processes on the community assembly of lake protistan and fungal communities. This model predicts the relationship between ASV detection frequency and their relative abundance across the wider metacommunity and it proposes that abundant taxa are widely distributed since they are more likely to disperse by chance among sampling sites, while rare taxa are more susceptible to loss among sampling sites through ecological drift. In this model, the immigration rate (m) represents the probability that a random loss of an individual taxon from a local community will be replaced by dispersal from the metacommunity. Model fit to the data is indicated by *R* ^2^ and 95% confidence intervals for all fit statistics were calculated using 1000 bootstrap replicates.

To quantify the relative roles of deterministic and stochastic processes in community assembly across each lake, we applied the biodiversity ecological null model as outlined by Stegen et al. (2013). The β‐nearest taxon index (βNTI) and the β‐mean nearest taxon distance (βMNTD) were calculated using the standardised ASV abundance table and the phylogenetic tree. The phylogenetic tree of the microbial communities was constructed by aligning sequences with Clustal Omega (Sievers et al. 2011) and inferring phylogeny using FastTree (Price et al. 2010). βNTI was determined as the difference between observed βMNTD and the null distribution. Values of βNTI > 2 or < −2 indicate deterministic processes (variable or homogeneous selection), while values within the range [2, −2] suggest that stochastic processes (homogenising dispersal, dispersal limitation) predominate. Additionally, Raup‐Crick (RC) beta diversity, calculated based on sequence abundance, was used to further differentiate stochastic processes (Chase et al. 2011). Assemblages were categorised as structured by dispersal limitation for RC values > 0.95, by homogenising dispersal for RC values < −0.95 and by random processes without a dominant factor for RC values within the range [−0.95, 0.95].

## 3 Results

### 3.1 Composition and Diversity of Protistan Communities and Functional Groups

**FIGURE 2:**
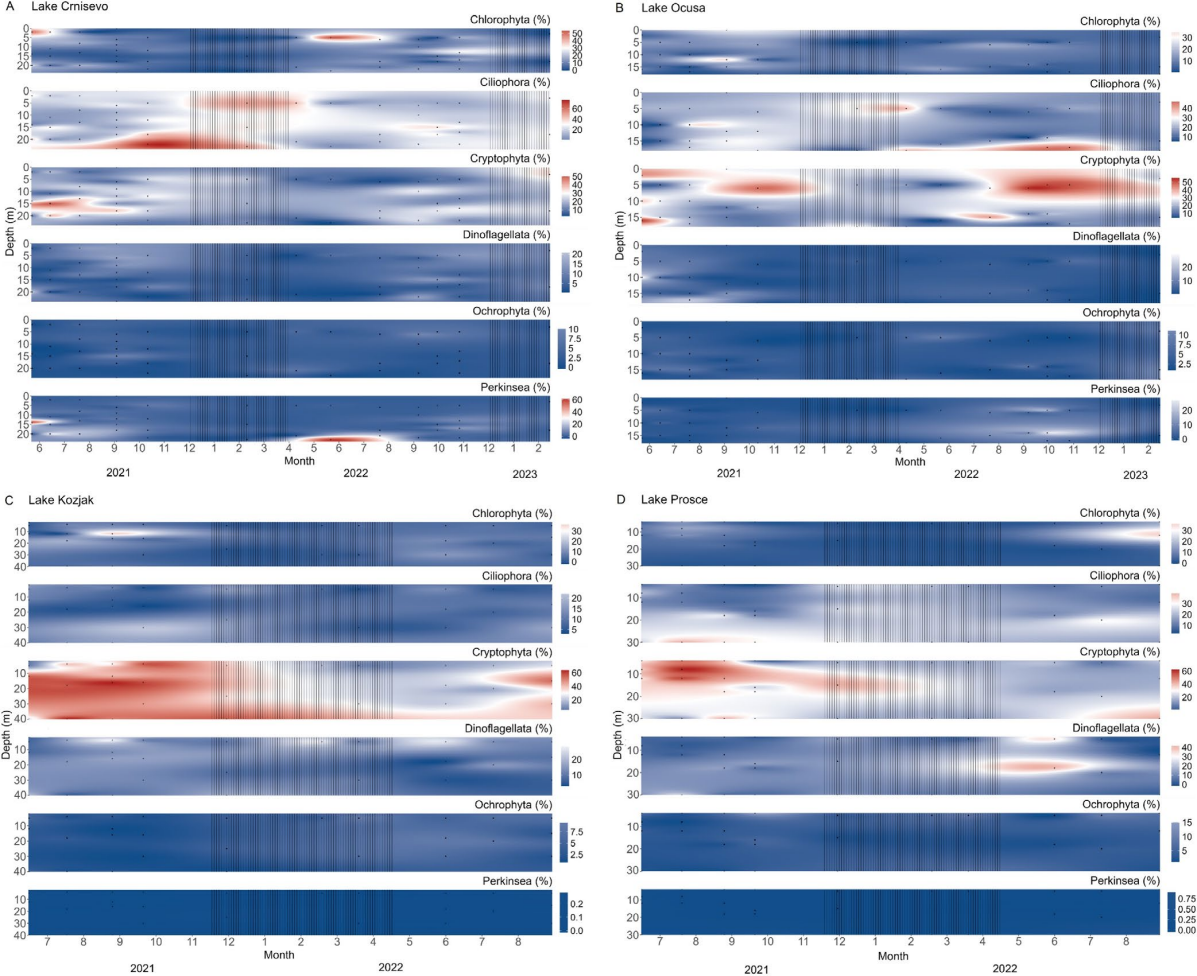
Distribution of the six most abundant protistan divisions in the whole community, based on 18S rRNA gene amplicon sequencing, throughout the sampling period in the studied lakes. (A) Crniševo; (B) Oćuša; (C) Kozjak; (D) Prošće. The lines represent mixing periods. Dots represent sampling points.

Figure 2 shows the distribution of the six most abundant protistan divisions throughout the sampling period in all four lakes. In Lake Crniševo, Chlorophyta, Ciliophora and Cryptophyta alternated in dominance and Perkinsea had high relative abundance in summer of all sampling years. Ciliophora and Cryptophyta were present throughout the sampling period, with Ciliophora peaking in abundance in autumn and winter months and Cryptophyta peaking in abundance in summer months. Chlorophyta peaked in abundance in surface layers in summer of all sampling years. In Lake Oćuša, Ciliophora and Cryptophyta alternated in dominance throughout the sampling period, with Ciliophora peaking in abundance during the late summer to early autumn of the first sampling year and again from spring through winter in the second sampling year, with the highest abundances observed around October. Cryptophyta showed peak abundance mainly in the summer months across all sampling years. In Lake Kozjak, Cryptophyta were the most dominant division throughout all depths in summer months and in the bottom layers in winter months, with Chlorophyta peaking in abundance in surface layers of the first sampling year. In Lake Prošće, Cryptophyta showed a similar pattern to that in Lake Kozjak and Chlorophyta peaked in surface waters, while Ciliophora had higher abundance throughout the entire sampling period. Also, Dinoflagellata were more abundant in Lake Prošće compared to the other three lakes, with peak abundance reported in June 2022.

**FIGURE 3:**
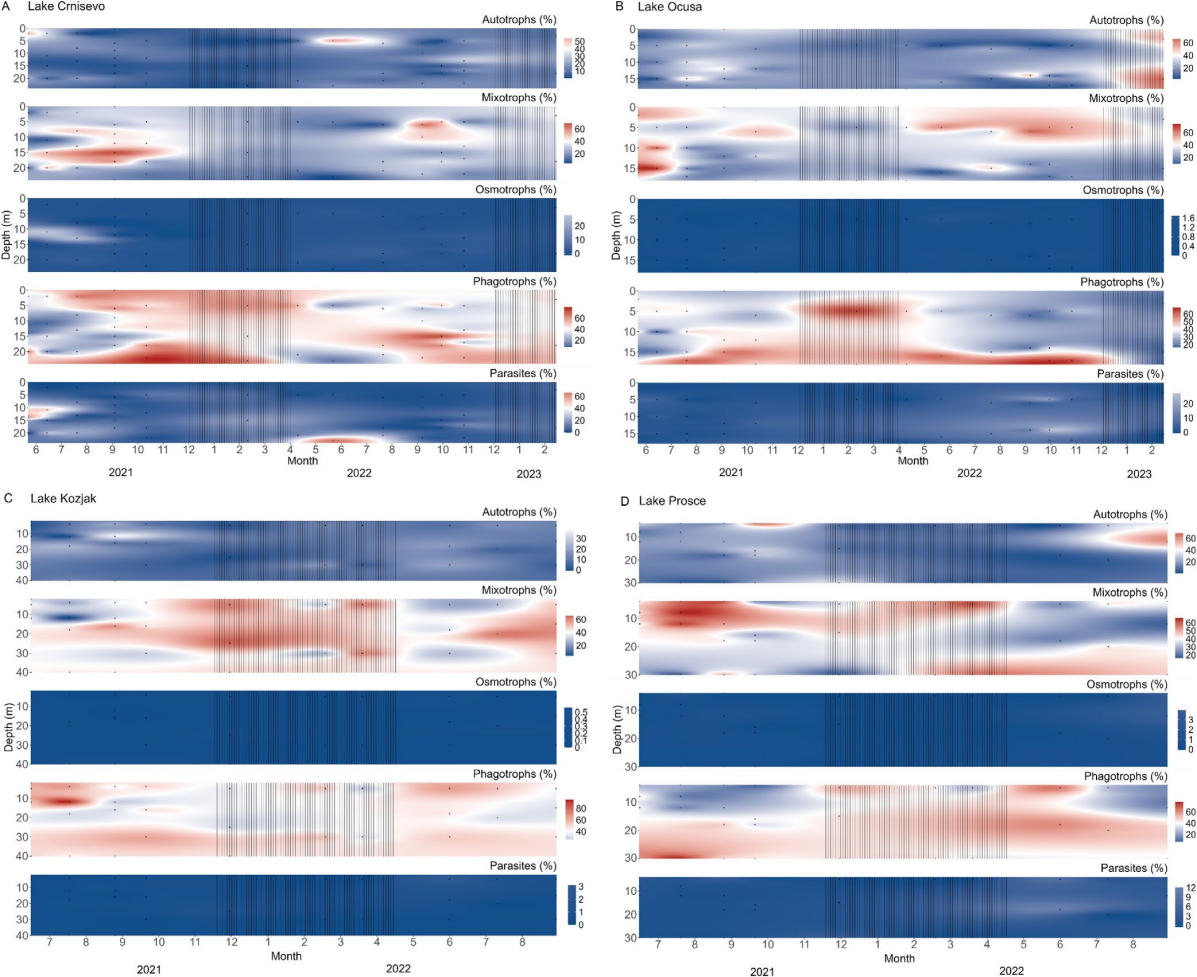
Distribution of the five most abundant protistan divisions in the whole community, based on 18S rRNA gene amplicon sequencing, throughout the sampling period in the studied lakes. (A) Crniševo; (B) Oćuša; (C) Kozjak; (D) Prošće. The lines represent mixing periods. Dots represent sampling points.

Figure 3 shows the distribution of the five functional groups throughout the sampling period in all four lakes. In Lake Crniševo, the most abundant were phagotrophs present throughout all depths but alternating in dominance with mixotrophs that peaked in abundance from summer to autumn months. Parasites were reported in high abundance in middle and lower depths in the summer months of all sampling years. Also, osmotrophs had the highest relative abundance in middle depths in the summer months of the first sampling year, 10‐fold higher than in any of the other lakes. In Lake Oćuša, phagotrophs were generally dominant throughout the year, especially in deeper waters where their highest abundances occurred from summer to autumn. In the upper water layers, phagotrophs peaked during winter and in spring. Mixotrophs showed a peak in summer 2021 and from spring to winter in 2022, but only in surface to middle layers. Overall, autotrophs had higher relative abundance across depths compared to Lake Crniševo. In Lakes Kozjak and Prošće, alternating dominance between phagotrophs and mixotrophs was reported. In Lake Kozjak, mixotrophs dominated the middle layer during summer months and the entire water column during winter, as well as in spring and autumn months of 2022. In contrast, phagotrophs occupied niches opposite to those of mixotrophs. In Lake Prošće, phagotrophs dominated most of the water column except for the upper layers from summer through autumn. Mixotrophs showed an opposing pattern to Lake Kozjak, with higher abundance in the upper layers in 2021 and bottom layers in 2022. Autotrophs were also more abundant in Lake Prošće compared to Lake Kozjak, peaking in the upper layers during summer months.

### 3.2 Composition and Diversity of Fungal Communities

**FIGURE 4:**
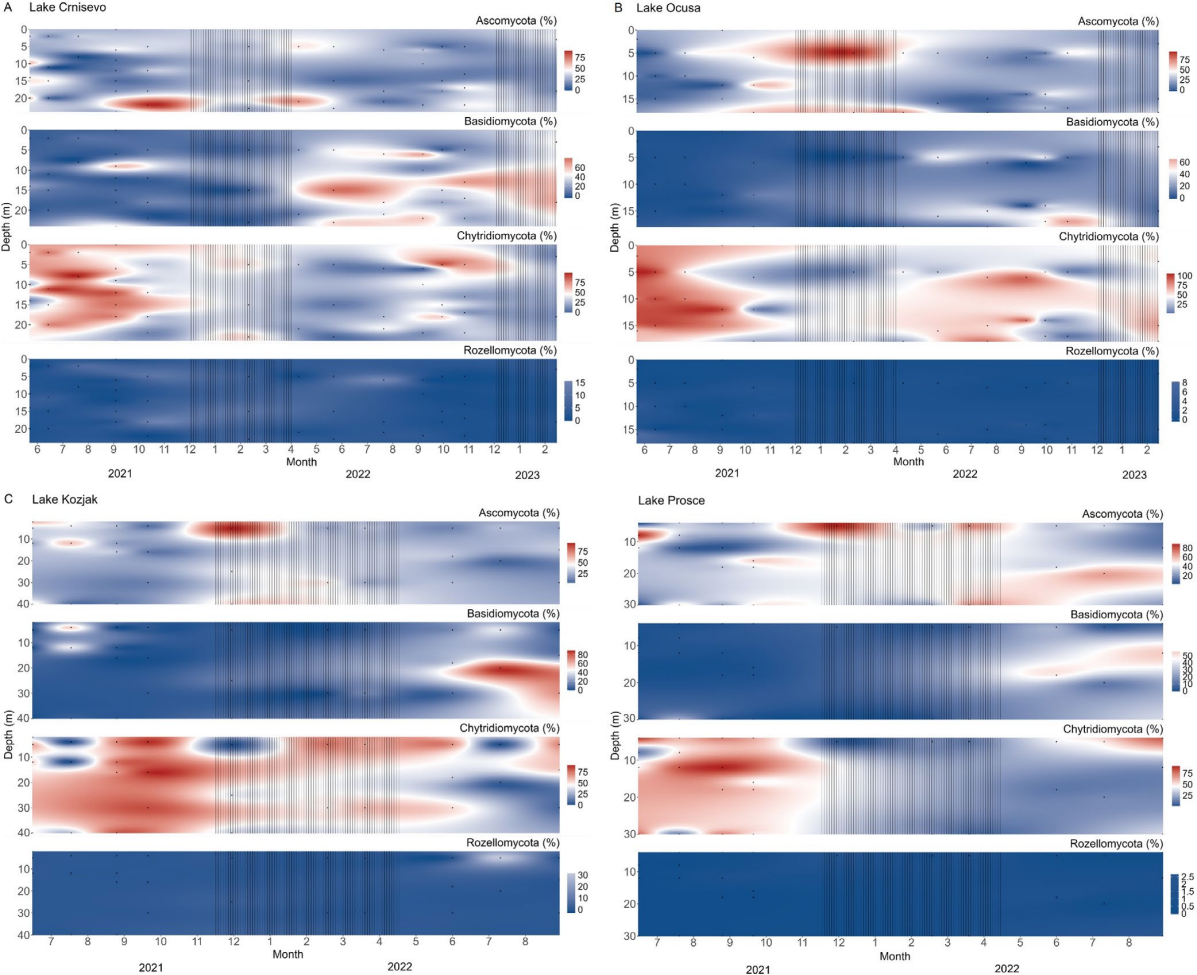
Distribution of the four most abundant fungal phyla in the whole community, based on ITS gene amplicon sequencing, throughout the sampling period in the studied lakes. (A) Crniševo; (B) Oćuša; (C) Kozjak; (D) Prošće. The lines represent mixing periods. Dots represent sampling points.

Figure 4 shows the distribution of the four most abundant fungal phyla throughout the sampling period in four investigated lakes. All four lakes showed a pattern of alternating dominance between Ascomycota, Basidiomycota and Chytridiomycota, while Rozellomycota had lower relative abundance. In Lake Crniševo, Chytridiomycota displayed the highest abundance throughout the water column during 2021, except in the bottom layers during the autumn and winter months when Ascomycota peaked in abundance. Basidiomycota exhibited their highest abundance from spring to autumn of the second sampling year, primarily in the middle to bottom layers, while Chytridiomycota predominantly occupied the upper layers during this period. In Lake Oćuša, Chytridiomycota dominated the water column throughout most seasons of all sampling years and Ascomycota peaked in abundance during the winter months of the first sampling year. Basidiomycota dominated the bottom part of the column in autumn 2022. In Lake Kozjak, Chytridiomycota was the most abundant phylum throughout the sampling period, with the exception of the upper layers of the water column in winter 2021, when Ascomycota peaked in abundance and the middle layers of the water column in summer 2022, when Basidiomycota peaked in abundance. The highest abundance of Rozellomycota in all four lakes was reported in the upper layer of Lake Kozjak in summer 2022. In Lake Prošće, Chytridiomycota dominated the entire water column from summer through autumn in 2021 and were also abundant in the upper layer during summer 2022. During the winter months, Ascomycota had the highest abundance, while Basidiomycota dominated the middle layer in summer 2022.

### 3.3 Stability of Fungal and Protistan Communities

**FIGURE 5:**
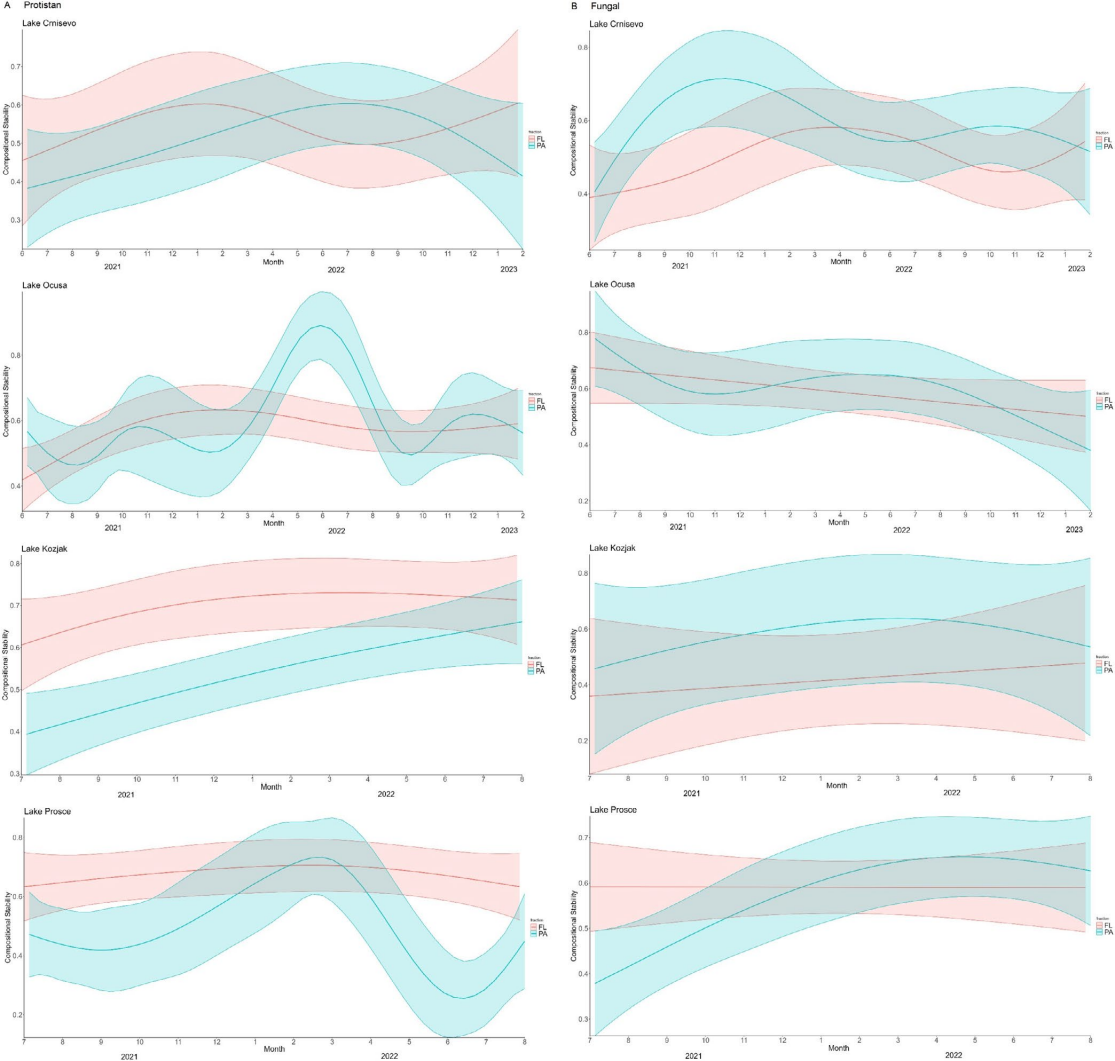
Compositional stability trends of (A) protistan and (B) fungal communities in four investigated lakes. Blue line represents particle‐associated fraction (PA) of the microbial community and red line represents free‐living fraction (FL) of the microbial community.

Compositional stability trends of protistan and fungal communities in four investigated lakes are shown in Figure 5. Similar average stability indices for protistan and fungal communities across the sampling period were reported in lakes Crniševo, Oćuša and Prošće, while in Lake Kozjak, protistan communities had a higher average stability index (0.6129) compared to fungal communities (0.4879). For protistan communities, the average stability index was highest in Lake Kozjak, especially for the FL fraction (0.6909) and lowest in Lake Crniševo (0.5192). For fungal communities, the average stability index was highest in Lake Oćuša, especially for the PA fraction (0.6086) and lowest in Lake Kozjak, especially for the FL fraction (0.4185). In Lake Crniševo, protistan FL communities had the highest value in winter and the lowest value in summer, while PA communities peaked in summer 2022. In Lake Oćuša, protistan FL communities peaked in winter 2021, while PA communities varied the most during the sampling period, reaching a peak in spring 2022. The stability of protistan FL communities in both Plitvice Lakes (Kozjak and Prošće) was stable throughout the sampling period. In Lake Kozjak, the stability of PA communities was rising throughout the sampling period, while in Lake Prošće it was more dynamic, peaking in winter 2022, then reaching the lowest value in summer 2022. Fungal communities followed similar trends. In Lake Crniševo, both the fungal PA and FL communities had the lowest stability in summer 2021, with PA peaking in winter 2021 and FL peaking in spring 2022. In Lake Oćuša, the stability of fungal FL communities was falling throughout the sampling period and PA communities were peaking in summer and sinking in winter months. In Plitvice Lakes, the stability of fungal FL communities was stable throughout the sampling period, while the stability of PA communities was rising, peaking in April 2022 in both lakes.

### 3.4 Community Assembly Processes

**FIGURE 6:**
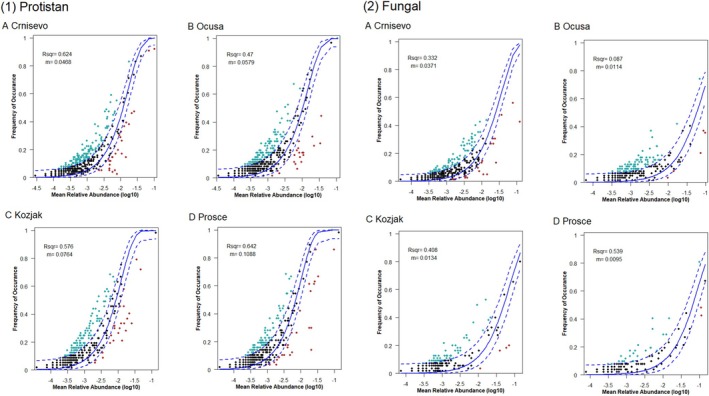
Neutral community model of (1) protistan and (2) fungal communities for investigated lakes (A—Crniševo, B—Oćuša, C—Kozjak, D—Prošće). The solid blue lines shows the best fit to the NCM, and the dashed blue lines represented 95% confidence intervals around the model prediction. Points indicate the OTUs, which were coloured differently depending on their occurrence frequency (red, black and blue points denote the below, neutral and above partitions, respectively). Rsqr is *R* ^2^ values and *m* is migration rate.

The neutral community model (NCM) showed that the neutral processes explain 59.7% of the protistan community variance and 39.3% of the fungal community variance. Analysing lakes separately, *R* ^2^ values ranged from 0.470 in Lake Oćuša to 0.642 in Lake Prošće for protistan communities and from 0.087 in Lake Oćuša to 0.539 in Lake Prošće for fungal communities (Figure 6). The estimated migration rate (m) was 0.0453 for protistan communities and 0.0115 for fungal communities (Figure 6). Lake Crniševo had the lowest estimated migration rate value for fungal communities and the highest for protistan communities, compared to other lakes.

**FIGURE 7:**
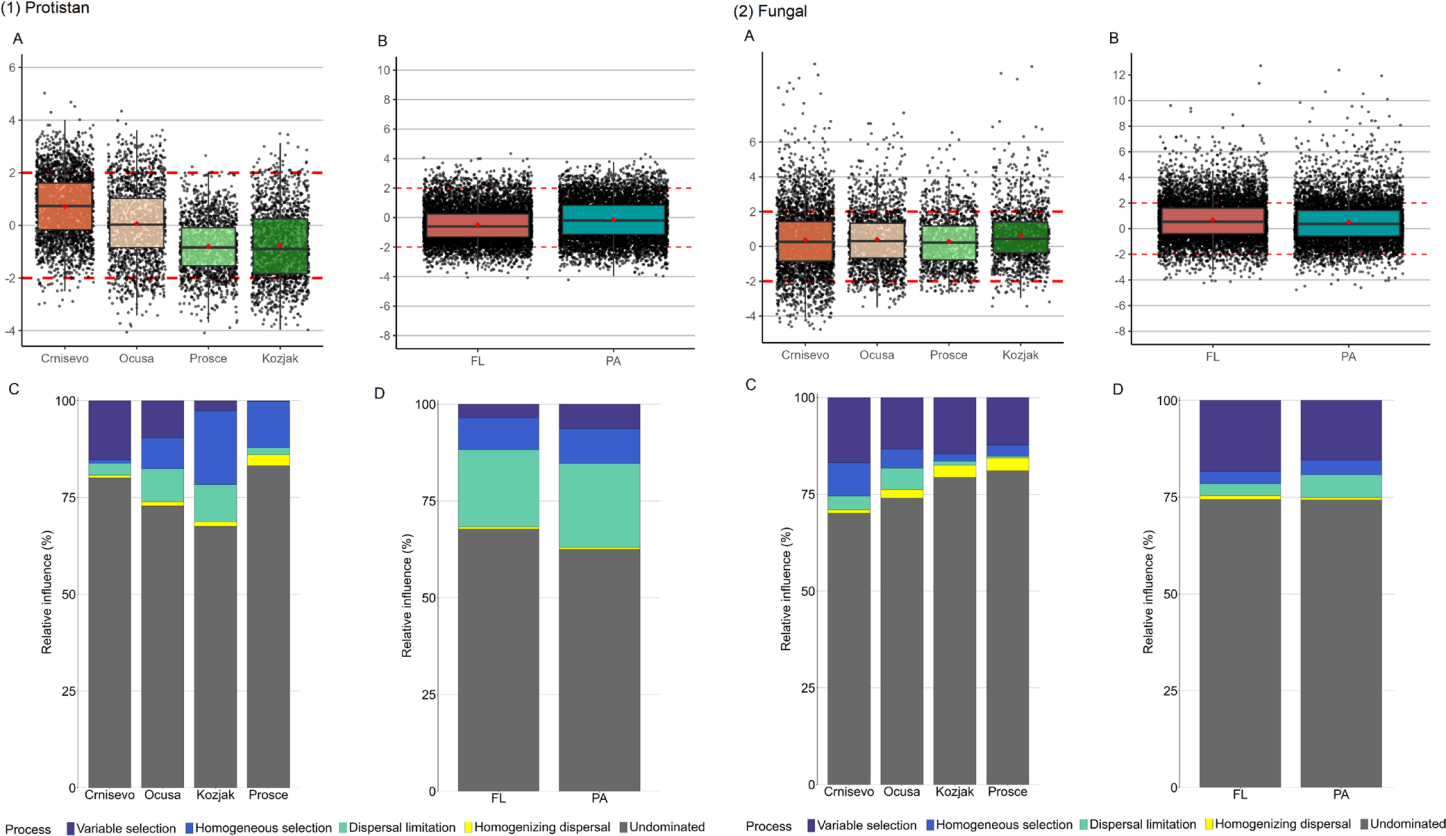
Null model analysis for (1) protistan and (2) fungal communities for investigated lakes (A, C) and fractions (B, D). Panels (A and B) show boxplots of the observed Bray–Curtis β‐nearest taxon indices for different lakes and fractions. Values > +2 or < −2 indicate significant influence of variable or homogeneous selection, respectively (indicated by red dashed lines). Values between −2 and +2 suggest stochastic processes or undominated assembly. Panels (C and D) depict the relative contributions of five ecological processes to community assembly, inferred using a null model approach combining βNTI with additional metrics (Raup–Crick).

Null model analysis showed that stochastic processes dominated most of the prokaryotic and fungal communities across sampled lakes and fractions, as mean βNTI values were between −2 and 2 (Figure 7). Undominated processes shaped the majority of both protistan and fungal communities. For protistan communities, variable selection had more influence on community assembly in Baćina Lakes, while homogeneous selection had more influence in Plitvice Lakes. For fungal communities, variable selection was the second most influential process across sampled lakes and fractions, while protistan FL and PA communities were equally influenced by dispersal limitation.

### 3.5 Partial Least Square Path Modelling

**FIGURE 8:**
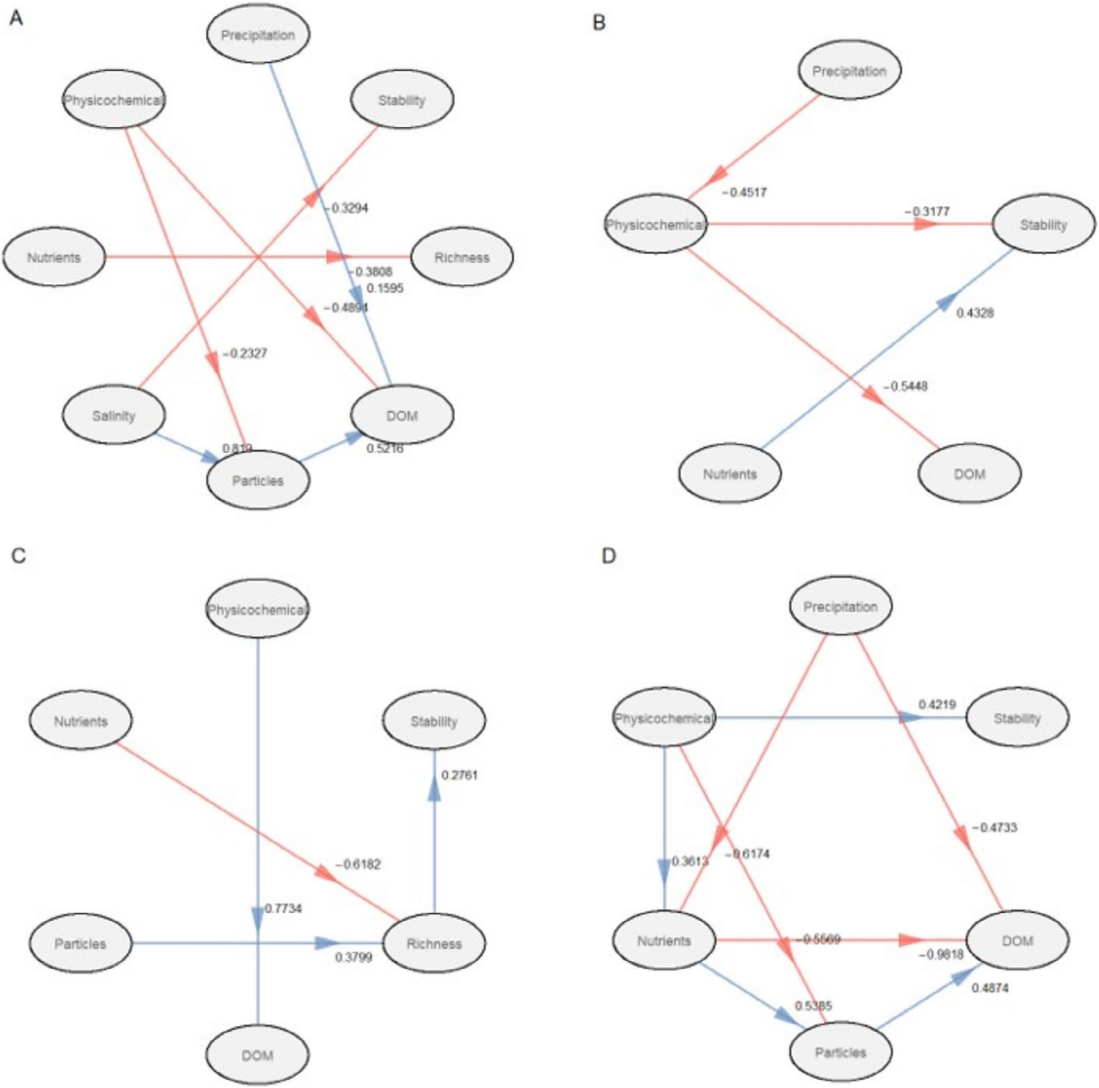
Partial least squares path models (PLS‐PM) showing the relationship between environmental drivers and the protistan community stability in four investigated lakes. Blue and red lines represent positive and negative effects, respectively, and only significant paths (*p* < 0.1) are shown.

**FIGURE 9:**
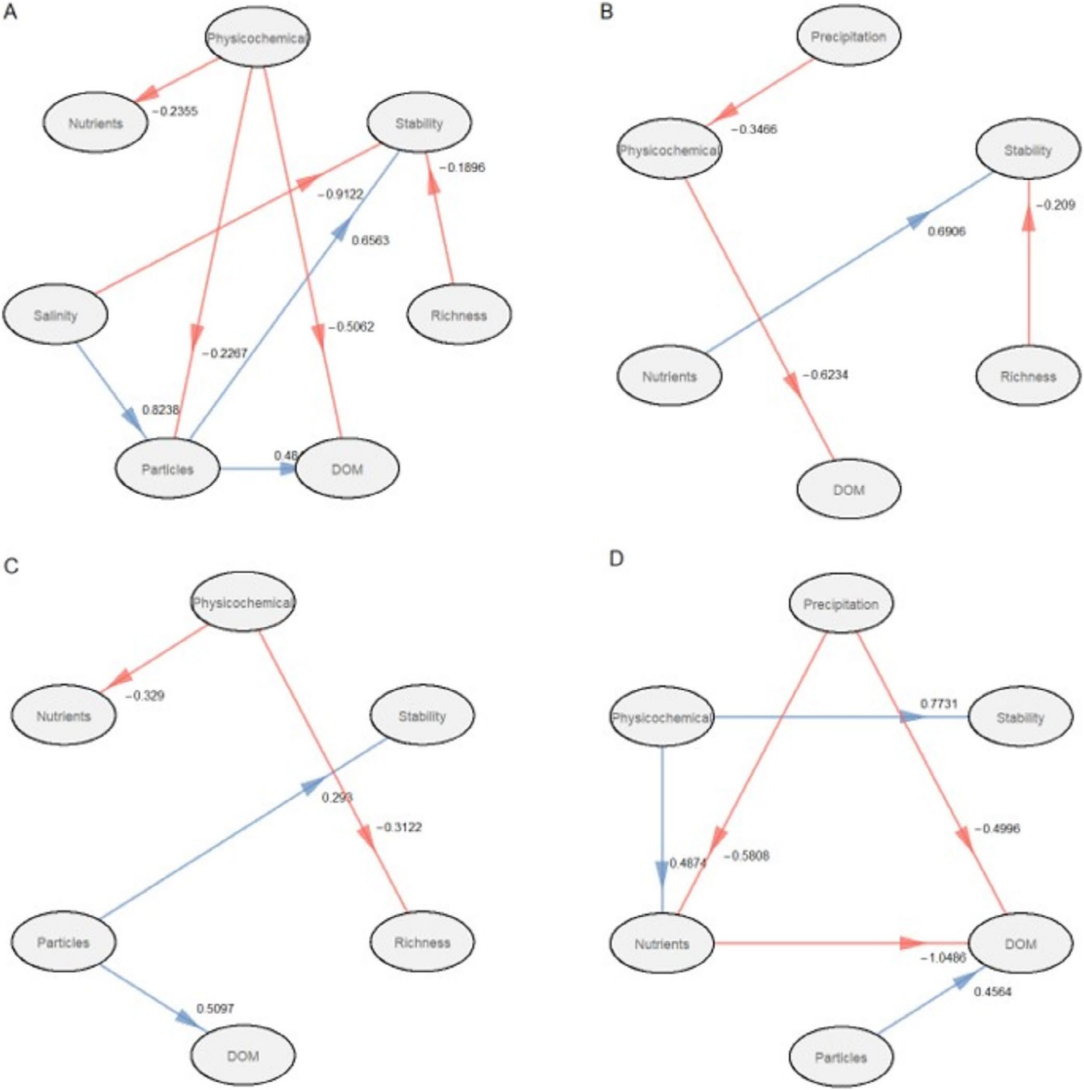
Partial least squares path models (PLS‐PM) showing the relationship between environmental drivers and the fungal community stability in four investigated lakes. Blue and red lines represent positive and negative effects, respectively, and only significant paths (*p* < 0.1) are shown.

The partial least square path modelling revealed direct and indirect effects of abiotic and biotic factors on the compositional stability of protistan and fungal communities across the four studied lakes (Figures 8 and 9). The correlations presented in the figures are statistically significant (*p* < 0.1). Goodness of fit was high for all lakes: 0.5459 for Lake Crniševo, 0.3311 for Lake Oćuša, 0.3939 for Lake Kozjak and 0.4547 for Lake Prošće. In Lake Crniševo, salinity had a negative effect (*r* = −0.3294, *p* < 0.1) on the stability of protistan communities, while in Lake Oćuša, nutrients had a positive effect (*r* = 0.4328, *p* < 0.1) and physicochemical parameters had a negative effect (*r* = −0.3177, *p* < 0.1). In Lake Kozjak, richness had a direct positive effect (*r* = 0.2761, *p* < 0.1), while nutrients and particles had an indirect effect on protistan community stability, negative and positive, respectively. In Lake Prošće, physicochemical parameters had a positive effect on the stability of the protistan community (*r* = 0.4219, *p* < 0.1). In Lake Crniševo, salinity and richness had a negative effect (*r* = −0.9122, *p* < 0.1 and *r* = −0.1896, *p* < 0.1, respectively) and particles had a positive effect (*r* = 0.6583, *p* < 0.1) on fungal community stability. In Lake Oćuša, nutrients had a positive effect (*r* = 0.6906, *p* < 0.1) and richness had a negative effect (*r* = −0.2090, *p* < 0.1) on fungal community stability. Particles had a positive effect (*r* = 0.2990, *p* < 0.1) in Lake Kozjak and physicochemical parameters had a positive effect (*r* = 0.7731, *p* < 0.1) in Lake Prošće on fungal community stability.

## 4 Discussion

### 4.1 Protistan and Fungal Community Composition and Distribution in Investigated Lakes

The protistan communities across the four studied lakes displayed distinct spatiotemporal variations in composition and functional groups, driven by seasonal changes, nutrient levels and depth stratification. Dominance alternated among key protistan divisions, including Chlorophyta, Ciliophora, Cryptophyta and Perkinsea, with specific trends linked to environmental conditions such as seasonal changes and nutrient availability. The higher relative abundance of Chlorophyta in surface layers, particularly in lakes Crniševo and Kozjak, aligns with their phototrophic nature and increased light availability in this layer. In contrast, Ciliophora and Cryptophyta, which have diverse trophic modes, exhibited more consistent presence throughout the year, reflecting their ecological versatility (Lin et al. 2022). Perkinsea, a group associated with parasitic lifestyles, showed high relative abundance during summer in Lake Crniševo, potentially driven by warmer temperatures that promote parasitic interactions (Lefèvre et al. 2011). The distinct seasonal peaks of functional groups were reported, such as phagotrophs dominating in autumn and winter in Baćina Lakes, while in Plitvice Lakes their highest relative abundances appeared during spring and summer months. This season‐specific dominance highlights the variable ecological roles of phagotrophs across lakes and seasons. In contrast, mixotrophs dominate in summer as they combine autotrophic and heterotrophic feeding modes, which allows them to utilise higher light availability and primary production during this period. Their ability to photosynthesise gives them a competitive advantage when resources like prey availability fluctuate. Heterotrophic protists tended to be more abundant in deeper, darker waters, while mixotrophs often peaked in upper to mid‐depth layers. This pattern aligns with the dependence of mixotrophs on light for photosynthesis in addition to organic resources, whereas heterotrophs can thrive in low‐light or dark conditions by consuming prey. Such depth‐dependent distribution of trophic groups reflects their adaptation to different ecological niches shaped by environmental gradients (Hansson et al. 2019). The exceptionally high abundance of osmotrophs in Lake Crniševo during the first summer sampling year suggests specific ecological niche in this lake. Higher salinity often leads to increased osmotic stress, which can reduce competition from other microbial groups less adapted to such environments. Osmotrophs, which absorb dissolved nutrients directly from their surroundings, are adapted to high salinity conditions. Also, depth‐specific differences in protistan community composition were observed, specifically in Oćuša between May and December. Phagotrophs were more abundant in bottom waters, while mixotrophic groups dominated the surface layers. This vertical separation likely reflects niche differentiation driven by varying environmental conditions, such as light availability and nutrient gradients, supporting the coexistence of diverse functional groups within the lake ecosystem (Fischer et al. 2022).

The alternating dominance of Ascomycota, Basidiomycota and Chytridiomycota over time and depth underscores their ecological specialisation and responses to environmental gradients. The high abundance of Chytridiomycota in surface layers during summer months, particularly in Lake Crniševo, aligns with their known roles as saprotrophs or parasites of phytoplankton, likely thriving on decaying organic matter or algal hosts (Gleason et al. 2008). These activities contribute significantly to nutrient cycling and organic matter turnover in aquatic ecosystems, supporting broader food web dynamics. The seasonal dominance of Ascomycota and Basidiomycota suggests their roles in organic matter degradation during cooler months, where slower microbial activity might allow fungal communities to dominate. These fungi likely play critical roles in breaking down complex organic compounds, facilitating the release of nutrients that sustain microbial and protistan communities. The peak abundance of Rozellomycota in the upper layers of Lake Kozjak in summer indicates potential interactions with specific hosts. During summer, the upper layers are often characterised by increased primary production, leading to an abundance of potential hosts and organic substrates. Rozellomycota, often parasitic or saprotrophic, may thrive in these conditions by targeting the high densities of algae and other microorganisms present in the photic zone (Quandt et al. 2017).

### 4.2 Community Stability and Partial Least Square Path Modelling

The stability of protistan and fungal communities varied across lakes and fractions, with Lake Kozjak exhibiting the highest stability index for protistan FL communities. This suggests that the oligotrophic nature, characterised by low nutrient concentrations and clear waters and stable environmental conditions of this lake may support more resilient protistan communities. These conditions might favour protists that thrive in low‐nutrient environments due to their diverse trophic strategies and adaptability. In contrast, the lower stability of fungal FL communities in Lake Kozjak highlights their potential sensitivity to nutrient limitation or competition with other microbial groups, which may hinder their ability to maintain stable populations under such conditions (Shade et al. 2012). The lowest stability for protistan communities was observed in Lake Crniševo, suggesting that salinity exerts a strong selective pressure, reducing stability due to osmotic stress and variability in adaptive capacities (Oren 2010). For fungal communities, the highest stability was recorded in Lake Oćuša, especially for particle‐associated (PA) fractions (0.6086). This could reflect the role of particulate organic matter as a stable substrate that supports fungal colonisation and growth (Grossart et al. 2019).

In Lake Crniševo, the seasonal peak of protistan FL stability during winter aligns with reduced competition and predation in colder months, as well as lower metabolic activity, which can stabilise populations. The summer peak of PA communities in 2022 may be driven by increased availability of organic particles and nutrient inputs during stratified conditions. For fungal communities in Lake Crniševo, summer 2021 exhibited the lowest stability, potentially due to heightened salinity stress and limited substrate availability. Peaks in PA fungal stability during winter further underscore the importance of particulate matter during periods of limited primary productivity. In Lake Oćuša, the dynamic variation in protistan PA stability, peaking in spring 2022, highlights the influence of seasonal nutrient turnover and organic particle fluxes, whereas fungal PA communities peaked during summer months, likely benefiting from increased particulate matter availability during these periods. Declining stability in fungal FL communities suggests increasing resource competition or environmental stress over time. For the Plitvice Lakes, stability trends suggest more consistent environmental conditions. FL fractions of protistan and fungal communities maintained stable indices across the sampling period, reflecting reduced variability in abiotic factors. The rising stability of PA fractions of both protistan and fungal communities, peaking in April 2022, indicates progressive enrichment of particulate substrates that may have supported community stability (Grossart et al. 2019).

The PLS‐PM analysis revealed complex direct and indirect effects of abiotic and biotic factors on community stability. In Lake Crniševo, salinity had a significant negative impact on protistan community stability, consistent with its role as a major selective pressure in saline environments. High salinity imposes osmotic stress, which can destabilise protistan populations by limiting the abundance of taxa adapted to such extreme conditions (Oren 2010). Similarly, salinity negatively affected fungal community stability, likely due to fungi's reliance on organic substrates and limited physiological mechanisms for coping with salinity (Grossart et al. 2019). The negative effect of richness on fungal stability in Lake Crniševo may reflect competitive exclusion among fungal taxa, where higher richness creates ecological pressures that reduce overall stability. A strong positive effect of particles on fungal stability highlights the importance of particulate organic matter as a stable substrate for fungal colonisation and growth in saline conditions (Grossart et al. 2019). In Lake Oćuša, nutrient enrichment likely enhances resource availability, supporting community resilience by facilitating growth and metabolic activity (Shade et al. 2012), explaining the positive effect on both protistan and fungal communities. The negative effect of physicochemical parameters on protistan stability could reflect fluctuations in temperature or oxygen levels, which can disrupt protistan population dynamics. The negative effect of richness on fungal stability might indicate that higher fungal diversity leads to increased interspecific competition or functional redundancy, which could reduce overall community coherence (Peay et al. 2016). In Lake Kozjak, richness having a direct positive effect on protistan community stability aligns with the idea that higher biodiversity enhances functional redundancy and buffering capacity, promoting resilience to environmental fluctuations (Loreau and de Mazancourt 2013). Particles' positive effect on fungal community stability emphasised the role of particulate substrates in providing a consistent resource base for fungal colonisation in this oligotrophic lake (Grossart et al. 2019). In Lake Prošće, a strong positive effect on community stability suggests that the relatively stable and oligotrophic conditions of Lake Prošće provide a good environment for both protistan and fungal communities to maintain compositional stability over time (Stegen et al. 2012).

### 4.3 Assembly Processes of Protistan and Fungal Communities

The neutral community model (NCM) indicated that stochastic processes were more influential for protistan communities (59.7%) compared to fungal communities (39.3%). The higher migration rate observed for protistan communities suggests greater dispersal potential compared to fungal communities. This higher dispersal potential can be attributed to the smaller size and motility of many protistan species, enabling them to move more effectively through water currents and across spatial gradients. Additionally, protists can form resistant cysts or other dispersal stages, which facilitate their transport between habitats (Li et al. 2022). In contrast, the lower migration rate observed for fungal communities highlights their reliance on local environmental conditions and inherent dispersal limitations. Aquatic fungi often exhibit a strong dependence on specific organic substrates or host organisms for growth and reproduction (Grossart et al. 2019). This dependence makes them more constrained to environments where suitable resources are available. Additionally, fungal dispersal mechanisms in aquatic ecosystems are generally less efficient compared to protists. While some fungi produce spores that can disperse passively, these spores often rely on hosts for transport, limiting their range (Grossart et al. 2019). These factors, combined with potential barriers like stratification or nutrient gradients, emphasise fungi's reliance on localised environmental stability and resource availability. Consequently, fungal communities tend to exhibit more spatially distinct populations and lower migration rates compared to free‐living protistan communities. This reduced dispersal capacity contributes to their sensitivity to environmental changes and highlights the importance of local ecological dynamics in shaping fungal distributions.

Null model analysis further supported the dominance of stochastic processes in shaping both protistan and fungal communities, with undominated processes prevailing in most cases. However, variable selection was more prominent in Baćina Lakes, particularly in Lake Crniševo, due to the salinity gradient. Such gradient creates diverse microhabitats and selective pressures that promote variable selection. This process favours microbial taxa that are highly specialised for specific niches or environmental conditions, leading to greater community differentiation across space and time (Oren 2010; Shade et al. 2012). In contrast, the Plitvice Lakes are oligotrophic, characterised by low nutrient concentrations, clear waters and more stable environmental conditions. This environmental stability reduces the strength of selection pressures and promotes homogeneous selection, where communities converge toward similar compositions regardless of spatial or temporal variations (Stegen et al. 2012).

## 5 Conclusions

This study provides important insights into the composition, stability and assembly mechanisms of protistan and fungal communities in freshwater lake ecosystems, highlighting their ecological roles and responses to environmental gradients. The findings underscore the dominance of stochastic processes in community assembly across most lakes and fractions, suggesting that random dispersal and ecological drift are key drivers of microbial community dynamics in these systems. However, in Lake Crniševo, a pronounced salinity gradient introduced strong variable selection, differentiating its community assembly from the other lakes. Salinity emerged as a critical environmental factor negatively impacting the stability of both protistan and fungal communities, reducing resilience and increasing community differentiation. These results highlight the susceptibility of microbial communities to salinity‐induced stress, particularly in seasonally salty environments like Lake Crniševo. By linking environmental factors to community composition and stability, this study advances our understanding of microbial dynamics in freshwater ecosystems and their responses to environmental changes. Moreover, light availability plays a key role in shaping the vertical distribution of protistan communities, with mixotrophic groups generally dominating the upper to mid‐depth layers where light supports photosynthesis, while heterotrophic groups prevail in deeper, darker waters. This vertical separation facilitates niche differentiation and contributes to overall community stability and functional diversity. These insights are crucial for the conservation and management of freshwater resources, especially under increasing anthropogenic pressures that may alter salinity and other critical environmental parameters. Understanding the interplay between stochastic and deterministic processes in microbial community assembly can inform strategies to maintain ecosystem services provided by freshwater lakes, ensuring their ecological balance and resilience in the face of global environmental change.

## Author Contributions

**Ivana Stanić:** investigation, writing – original draft, methodology, formal analysis. **Andrea Čačković:** investigation, visualization. **Sandi Orlić:** funding acquisition, writing – review and editing, project administration, supervision, conceptualization.

## Conflicts of Interest

The authors declare no conflicts of interest.

## Data Availability

The data that support the findings of this study are available upon request. Amplicon sequencing data are openly available in the NCBI repository under BioProject accession number PRJNA1224720.
